# Supplementary material for: An Intergenerational Exploration of Discipline, Attachment, and Black Mother–Daughter Relationships Across the Lifespan
Source: Behav Sci (Basel). 2025 Jun 30;15(7):887. doi: 10.3390/bs15070887 (PMC12292128; doi:10.3390/bs15070887)
Supplement: Supplementary file 1 [file behavsci-15-00887-s001.zip › behavsci-3547820-supplementary.pdf]

Part 1: Disciplinary Experiences during Childhood

So first, we're going to talk about your disciplinary experiences during childhood.

1. Let us start by talking about your own relationship with your mother. How would you describe your relationship?
2. From what you recall, what types of disciplinary styles did your mother use when you were younger?
3. What did you think about your mother's disciplinary style when you were a child?
4. What do you think about your mother's disciplinary style now, as a mother with children?
5. Tell me about where you grew up. Do you think the discipline you experienced was similar to other Black children in your neighborhood and/or school?

Part 2: Current Parenting Practices

Now, we're going to dive into your parenting practices.

1. Can you describe how you became a mother?
2. In the current study, we think of conscious parenting as guiding children through their emotional responses, refraining from physical or corporal punishment, and thinking through your personal triggers as a parent to engage with your child in thoughtful and supportive ways. But we want to know - what does conscious parenting mean to you?
3. Did you receive support from others?
4. Some people associate Black parents with harsher disciplinary practices – like spanking or yelling at their children. Are you aware of these stereotypes, and what do you think of them?

Part 3: Race-Related Beliefs and Experiences

Now, I want to talk about your experiences as a Black woman and as a Black mother. As a reminder, you are not required to answer any questions that you do not feel comfortable answering.

5. If at all, what messages did your parents give you about race? This could include messages about being a Black girl or how to handle discrimination.
6. In what ways do you plan to prepare your children to cope with racism?
7. In the age of social media, we are exposed to frequent stories of police brutality and violence against Black people and communities. To what extent do these stories influence you as a mother? How do you process them?

Part 4: Advice

Now, we're going to wrap up with advice you would give to other Black mothers or Black parents.

1. What advice would you give to Black mothers who want to use conscious parenting practices?
2. What do you wish you could tell yourself when you were just starting your parenting journey?
